# Supplementary material for: Low-Cost Self-Calibration Data Glove Based on Space-Division Multiplexed Flexible Optical Fiber Sensor
Source: Polymers (Basel). 2022 Sep 20;14(19):3935. doi: 10.3390/polym14193935 (PMC9572434; doi:10.3390/polym14193935)
Supplement: Supplementary file 1 [file polymers-14-03935-s001.zip › polymers-1863653-supplementary-r1/Supplementary material/Supplementary File.pdf]

1. All the components of this data glove were purchased at a very cheap price. The actual price of each component is recorded in Table S1.

Components	Price
**Textile gloves**	**$4.4**
**Cameras and light sources**	**$11.6**
**Materials for fabricating flexible optical fibers**	**$5.8**
**Total sum**	**$21.8 (Material cost, No labor cost)**

Table S1. The actual price of each component of the data glove.

2. There are many published data gloves, but they are expensive. Table S2 shows the price of data gloves for some different brands and models. For example, the 5dt data glove 14 ultra costs up to $5,495, with an additional charge for glove cleaning. The CaptoGlove glove is the cheapest looking glove available, but it's a tactile glove that doesn't track gestures, and at $440, it's still not that cheap. Of course, these commercial data gloves have been tested for reliability before entering the market. Our design is a long way from them. However, the cost of our data gloves is extremely low. The actual price of each component is recorded in Table S1. The cost of a single glove is less than $22. If we purchase raw materials in large quantities, it can be even cheaper.

Designs	Brand (Model)	Mechanism	Country	Price
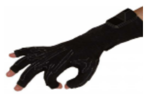	**5DT** (5dt data glove 14 ultra)	Fiber based bending sensor	**South Africa**	$5495
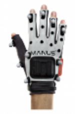	Manus (Manus Prime X Makers Mocap Light and habitus mixed gloves)	Optical inertial mixed sensor	Netherlands	$4990
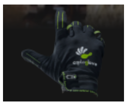	**CaptoGlove** (CaptoGlove Touch gloves)	Flex bending sensor	USA	$440
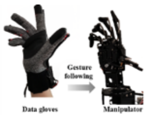	Our design	Flexible optical fiber strain sensor	China	$21.8 (Material cost, No labor cost)

Table S2. Price of data gloves of different brands and models.
